# FTO-dependent m6A methylation mediates gestational diabetes mellitus-induced offspring cardiac senescent hypertrophy and dysfunction

**DOI:** 10.1016/j.isci.2025.114311

**Published:** 2025-12-05

**Authors:** Wansu Yu, Yong Li, Siyi Jiang, Jia Tian, Shu-Wei Sun, Lubo Zhang, DaLiao Xiao

**Affiliations:** 1 Lawrence D. Longo, MD Center for Perinatal Biology, Department of Basic Sciences, Loma Linda University School of Medicine, Loma Linda, CA, USA; 2 Department of Geriatrics, Guangzhou First People’s Hospital, School of Medicine, South China University of Technology, Guangzhou, China; 3 Department of Hematology, The Third Xiangya Hospital of Central South University, Changsha, China

**Keywords:** molecular mechanism of gene regulation, epigenetics

## Abstract

Fat mass and obesity-associated (FTO) protein plays a critical role in N6-methyladenosine (m6A) demethylation, linked to metabolic disorders such as diabetes and obesity. This study investigates FTO-dependent m6A methylation in fetal programming of cardiac dysfunction due to gestational diabetes mellitus (GDM). Using a Sprague-Dawley rat model of GDM, we observed that GDM exposure repressed FTO, increasing m6A RNA methylation, consequently developing a cardiac hypertrophic dysfunctional phenotype in neonatal offspring. FTO inhibition replicated the effects of GDM, while overexpression of FTO via FTO lentivirus (Lenti-FTO) reversed GDM-induced hypertrophy, cardiac senescence, and dysfunction. These results illuminate the molecular mechanisms by which GDM negatively impacts offspring cardiac health and highlight the potential for targeting FTO-mediated RNA methylation pathways as a therapeutic strategy for GDM-related cardiac issues.

## Highlights

• GDM represses cardiac FTO, increasing m6A methylation and hypertrophy in offspring
• FTO-dependent m6A regulation links GDM to cardiac dysfunction in neonatal hearts
• FTO restoration alleviates GDM-induced cardiac senescence and mitochondrial defects
• Targeting the FTO/m6A axis may help prevent GDM-induced cardiac programming

## Graphical abstract

**Figure:**
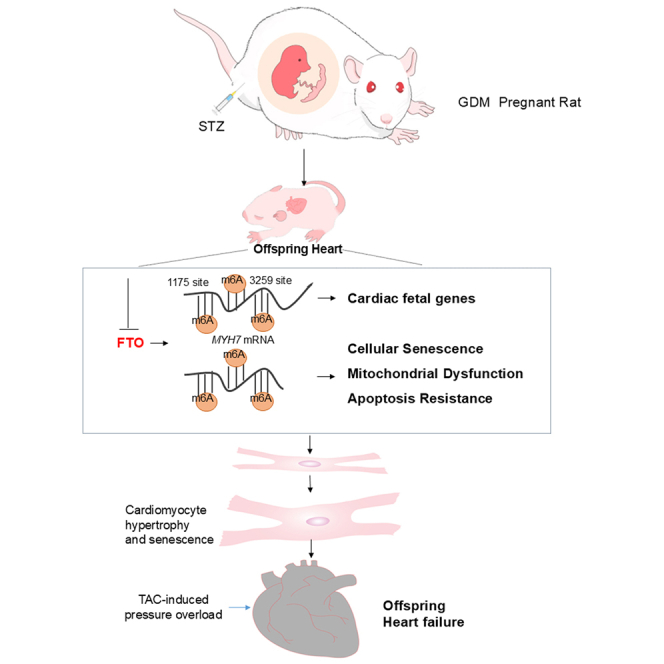

## Introduction

Gestational diabetes mellitus (GDM) stands as the most prevalent medical complication during pregnancy, characterized by glucose intolerance with onset or first recognition during gestation.^1^ Its prevalence ranges from 9% to 30% on average, soaring up to 31.5% in certain regions and countries worldwide.^2^^,^^3^ Recent evidence underscores the significant short- and long-term implications of GDM on cardiovascular health in offspring,^4^ yet the precise molecular mechanisms remain largely elusive.

Epidemiological and preclinical investigations have elucidated a positive association between GDM and fetal cardiac hypertrophy.^5^^,^^6^ While clinical presentations can vary, cardiac hypertrophy often appears early in infants born to mothers with gestational diabetes and may spontaneously resolve later in postnatal life, suggesting potential reversibility of this fetal cardiac adaptation.^7^^,^^8^ Cardiac hypertrophy typically serves as an adaptive response to cardiac stressors; however, its persistence can lead to adverse cardiac remodeling and eventual heart failure.^9^ Notably, cardiac fetal genes, including Myh7 (β-myosin heavy chain), Myh3 (coding embryonic myosin heavy chain), Nppa (natriuretic peptides A), and Nppb, play pivotal roles in heart development.^10^ The reactivation of these fetal genes, termed the “fetal gene program,” is a hallmark in the context of cardiac growth/hypertrophy and cardiomyopathy in postnatal life.^10^ Abnormal expression of fetal genes, such as Myh7 or Myh3, has been associated with cardiac hypertrophy during cardiac development.^11^^,^^12^

In addition to cardiac fetal genes, cellular senescence has also been proposed as a potential mechanism leading to cardiac hypertrophy in adult heart.^13^ In response to stress, mammalian cells undergo cellular senescence characterized by cellular proliferative arrest, apoptosis resistance, special secretory phenotype, increased cell size, heightened senescence-associated β-galactosidase (β-gal) activity, and reduced levels of cyclin-dependent kinase.^13^^,^^14^ While cellular senescence is well documented for its contribution to many age-associated diseases, its potential role in embryonic diseases and developmental defects remains underexplored.^15^^,^^16^ Furthermore, the precise regulatory mechanisms of cellular senescence in these conditions remain enigmatic.

Epigenetic modifications, including DNA methylation, histone modification, and non-coding RNAs, profoundly impact cardiovascular development. N6-methyladenosine (m6A) mRNA methylation has emerged as a widely distributed and significant epigenetic player implicated in cardiac hypertrophy development.^17^ This process is dynamically regulated by dedicated writers (methyltransferases) catalyzing m6A addition (METTL3, METTL14, and WTAP) and erasers (demethylases) facilitating m6A removal (FTO, ALKBH5) from mRNA.^18^^,^^19^^,^^20^ The fat mass and obesity-associated (FTO) protein, a pivotal player in m6A demethylation, has been linked to metabolic disorders such as diabetes and obesity.^21^ Notably, while FTO expression spans various tissues, its heightened levels in cardiac ventricles during human embryonic stages underscore its relevance.^17^ Despite its relevance, investigations examining the impact of maternal metabolic disorders such as GDM on FTO-dependent m6A demethylation and its consequent influence on cardiac development remain scarce.

The present study aims to elucidate the underlying mechanisms of cardiac dysfunction in GDM-exposed offspring. Initially, we employed a well-established pregnant rat model of GDM exposure to assess whether GDM instigated cardiac hypertrophy in offspring and led to the development of dysfunctional phenotype in postnatal life. Subsequently, we investigated whether GDM exposure augmented FTO-derived m6A RNA methylation, myocardial senescence accompanied by mitochondrial dysfunction, and resistance to apoptosis in offspring. Finally, gene therapeutic approaches were employed to examine whether the restoration of FTO gene expression via FTO lentivirus (Lenti-FTO) could reverse GDM-induced myocardial senescence, thereby rescuing GDM-induced cardiac hypertrophy and attenuating cardiac dysfunction in offspring.

## Results

### Impact of GDM exposure on cardiac function and TAC-induced heart failure

**Figure 1:**
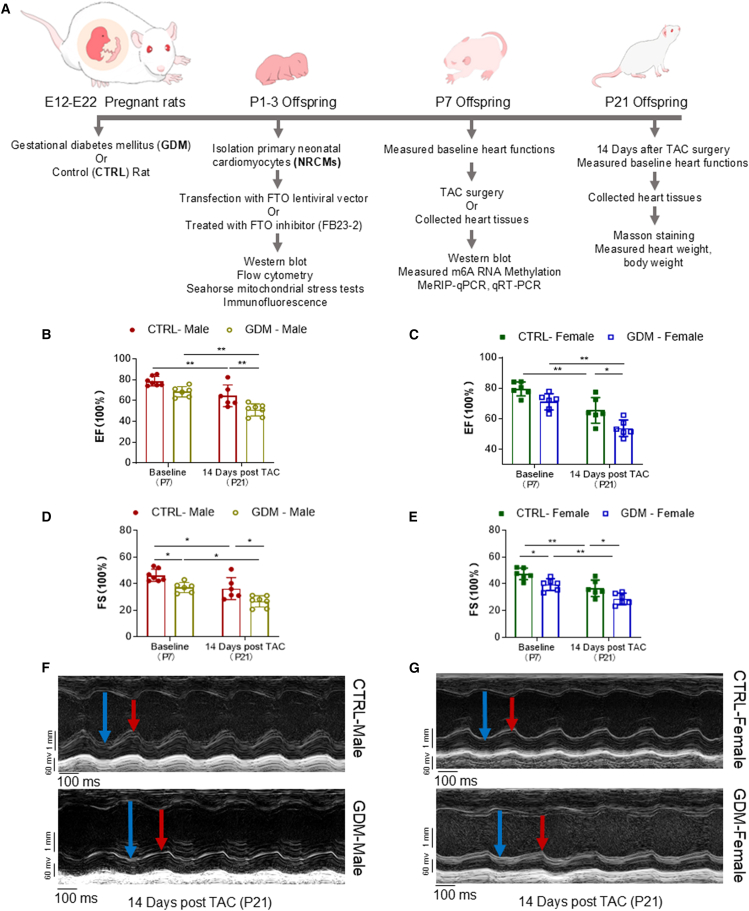
GDM exposure induces decreases in heart function in offspring (A) Experimental protocol. (B and C) Cardiac function ejection fraction (EF%) was examined by echocardiography at baseline before TAC (P7) and 14 days post TAC (P21) in male offspring (B) (CTRL,●; GDM,○) and female offspring (C) (CTRL,▪; GDM,□). (D and E) Fractional shortening (FS%) was examined in male offspring (D) (CTRL,●; GDM,○) and female offspring (E) (CTRL,▪; GDM,□). (F and G) Representative echocardiography evaluation of cardiac function in male (F) and female (G) offspring at 14 days post TAC. Data are expressed as means ± SD, *∗∗p* < 0.01 and *∗p* < 0.05 by two-way ANOVA followed by post hoc test. *n* = 7/group for baseline CTRL male group and 14 days post TAC GDM male group, *n* = 6/group for baseline GDM male group, 14 days post TAC CTRL male group and female groups.

To assess the influence of GDM on offspring cardiac function, echocardiography was performed on post-natal day 7 (P7) and post-natal day 21 (P21) following transverse aortic constriction (TAC) surgery. Before the TAC procedure on P7 offspring, GDM exposure had no significant effects on ejection fraction (EF%) in either male (Figure 1B) or female (Figure 1C) offspring compared to their respective sex-matched controls (CTRL). However, GDM exposure significantly reduced fractional shortening (FS%) in both male (Figure 1D) and female (Figure 1E) offspring and selectively attenuated left ventricular posterior wall thickness during systole (LVPWs) in P7 offspring (Figure S1B) compared to their respective CTRL.

Fourteen days after TAC procedure (P21), cardiac function, including ejection fraction (EF%) and fractional shortening (FS%), was significantly decreased in both male (Figures 1B and 1D) and female (Figures 1C and 1E) CTRL offspring compared to their respective baseline values. These reductions in function were accompanied by significant increases in left ventricular chamber size-related parameters, including left ventricular end-systolic and end-diastolic diameters (LVSd, LVSs, LVPWd, LVPWs, LVIDd, and LVIDs), left ventricular volumes (LV Vol d and LV Vol s), and stroke volume (SV), in both male (Figure S1A) and female (Figure S1B) CTRL offspring compared to their respective baseline values. Similarly, TAC-induced reductions in cardiac function were associated with increased left ventricular chamber size-related parameters compared to the sham group (Table S1), suggesting that TAC can induce cardiac hypertrophic dysfunction in neonatal pups. Notably, GDM exposure exacerbated TAC-induced reductions in cardiac function in both male (Figures 1B and 1D) and female (Figures 1C and 1E) offspring. Furthermore, GDM exposure selectively attenuated TAC-induced increases in left ventricular chamber size-related parameters, including LVSd, LVSs, LVPWs, LV Vol d, and SV, in both male and female offspring (Figures S1A and S1B, respectively).

### GDM exposure and cardiac hypertrophy

**Figure 2:**
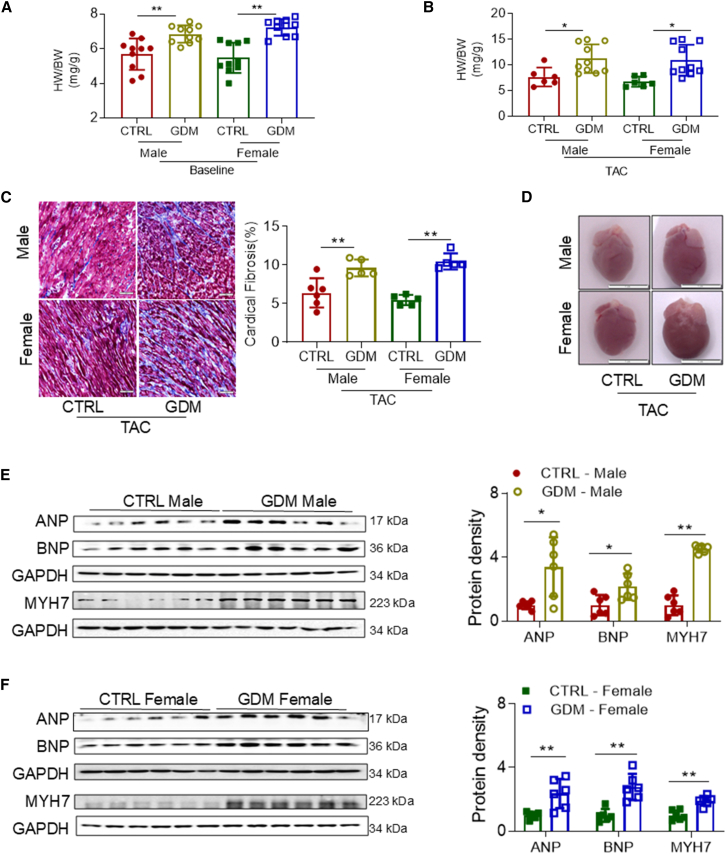
GDM exposure aggravates pathological cardiac hypertrophy in offspring (A) Heart weight/body weight (HW/BW) ratio was measured at baseline before TAC (P7) in male offspring (CTRL,●; GDM,○) and female offspring (CTRL,▪; GDM,□). *n* = 10/group. (B) HW/BW ratio was measured 14 days post TAC (P21) in male offspring (CTRL,●; GDM,○) and female offspring (CTRL,▪; GDM,□). *n* = 6/group for CTRL group and *n* = 10/group for GDM group. (C) Collagen in the cardiac tissues of the papillary muscles was examined at 14 days post TAC (P21) using Masson’s trichrome staining on paraffin-embedded sections for male offspring (CTRL,●; GDM,○) and female offspring (CTRL,▪; GDM,□). The scale bars represent 50 μm. *n* = 6/group for CTRL male group and *n* = 5/group for other groups. (D–F) (D) Representative gross photograph of the hearts in male and female offspring 14 days post TAC. The scale bars represent 1 cm. Pathological hypertrophy-related cardiac fetal genes at baseline before TAC (P7) were examined by western blotting in male offspring (CTRL,●; GDM,○) (E) and female offspring (CTRL,▪; GDM,□) (F). Data are expressed as means ± SD, *n* = 6/group. *∗∗p* < 0.01 and *∗p* < 0.05 by two-way ANOVA followed by post hoc test (A, B, and C) or Student’s two-tailed unpaired *t* test (E and F).

As shown in Figure 2A, GDM exposure significantly increased the heart weight/body weight (HW/BW) ratio in both male and female neonatal offspring compared to their respective CTRL. Following the TAC procedure, GDM-exposed offspring exhibited exacerbated cardiac hypertrophic responses and remodeling, as evidenced by significantly elevated HW/BW ratio in both male and female offspring (Figure 2B). Moreover, GDM exposure led to increased cardiac fibrosis in both male and female offspring (Figure 2C). Notably, gross photographs of hearts from GDM-exposed offspring post TAC showed a pronounced increase in heart size and a more rounded apex compared to their respective CTRL hearts (Figure 2D).

These structural and histological alterations in GDM-exposed offspring were associated with overexpression of pathological hypertrophy markers, including atrial natriuretic peptide (ANP), brain natriuretic peptide (BNP), and myosin heavy chain 7 (MYH7) at the protein level in both male (Figure 2E) and female offspring (Figure 2F). These findings suggest that GDM-induced cardiac hypertrophy may lead to pathological myocardial remodeling, contributing to cardiac dysfunction during the late stage of cardiac decompensation.

### GDM exposure and m6A RNA methylation

**Figure 3:**
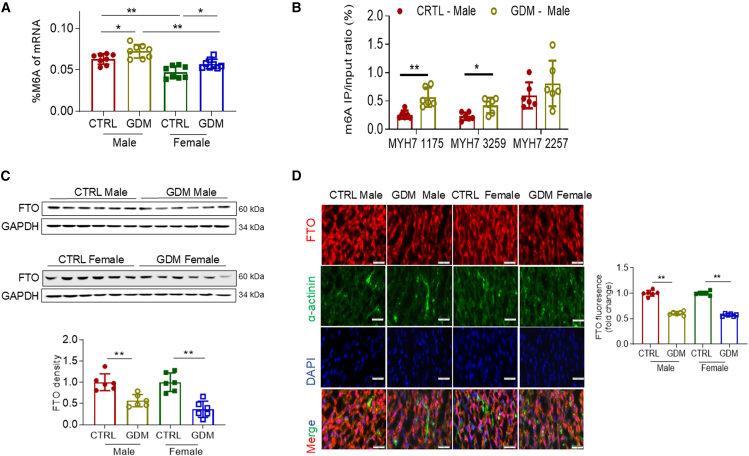
GDM exposure increases m6A RNA methylation associated with repression of FTO gene in offspring (A) Total levels of myocardial m6A RNA methylation were measured using the assay kits at baseline before TAC (P7) in male offspring (CTRL,●; GDM,○) and female offspring (CTRL,▪; GDM,□). *n* = 8/group. (B) The specific m6A methylation levels at positions 1175, 2257, and 3259 within the coding sequence (CDS) region, extending into the proximal 3′ untranslated region (3′ UTR) of MYH7 mRNA of male offspring heart, were determined by MeRIP-qPCR analysis as described in the STAR Methods. (C) The protein expression of FTO from heart tissue at baseline before TAC (P7) was examined using western blotting in male offspring (CTRL,●; GDM,○) and female offspring (CTRL,▪; GDM,□). *n* = 6/group. (D) FTO abundance was examined by immunofluorescence staining in frozen-embedded sections prepared from the papillary muscles of cardiac tissues at baseline before TAC (P7). α-Actinin was used to stain myocardium, and DAPI was used to counterstain the nuclei. The scale bars represent 50 μm. *n* = 6/group. Data are expressed as means ± SD, *∗∗p* < 0.01 and *∗p* < 0.05 by two-way ANOVA followed by post hoc test.

To investigate the impact of GDM exposure on RNA methylation (specifically m6A methylation) in the developing heart, we assessed the myocardial levels of total m6A methylation in both male and female offspring. As depicted in Figure 3A, GDM exposure significantly increased myocardial levels of total m6A methylation in both male and female offspring compared to their respective CTRL groups. Notably, the levels of m6A methylation were significantly higher in male offspring than in female offspring.

Building on these findings, we next examined specific m6A modification sites in the MYH7 gene, which could be involved in regulating gene expression. Using the sequence-based RNA adenosine methylation site predictor (http://www.cuilab.cn/sramp), we identified one high-confidence m6A site (site 1175) and two potential high-confidence m6A sites (sites 3259 and 2257) in the protein-coding sequence (CDS), extending into the proximal 3′ untranslated region (3′ UTR) of rat MYH7 mRNA (Figure 3B). To determine whether the increase in MYH7 expression observed with GDM exposure was regulated by m6A modification, we measured m6A levels in MYH7 mRNA using m6A immunoprecipitation (MeRIP) followed by RT-qPCR (MeRIP-qPCR). MeRIP-qPCR analysis (Figure 3B) revealed that GDM exposure significantly increased m6A levels at sites 1175 and 3259, but not at site 2257, in the CDS region of MYH7 in male offspring. These results suggest that changes in m6A at these specific sites are functionally relevant for regulating MYH7 expression in response to GDM exposure.

To explore potential mechanisms underlying the observed changes in m6A modification, we assessed the expression levels of m6A methyltransferases (METTL3 and WTAP) and the demethylase (FTO). Our data showed that GDM exposure did not alter the expression of METTL3 and WTAP (Figures S2A and S2B) but significantly decreased FTO protein levels (Figure 3C) in both male and female offspring compared to their respective CTRL. Additionally, because YTHDF2 is a known m6A reader that regulates target gene expression, we also measured cardiac levels of YTHDF2. Interestingly, GDM exposure did not affect YTHDF2 protein expression levels in male offspring (Figure S2C).

FTO is the first identified m6A demethylase in eukaryotic cells. The GDM-mediated repression of FTO was further confirmed by immunofluorescence staining, which revealed lower levels of FTO expression in the cardiomyocytes of myocardial tissue from GDM-exposed offspring compared to their respective CTRL in both male and female offspring (Figure 3D).

### Gene therapy with FTO lentivirus rescues GDM-induced hypertrophy

**Figure 4:**
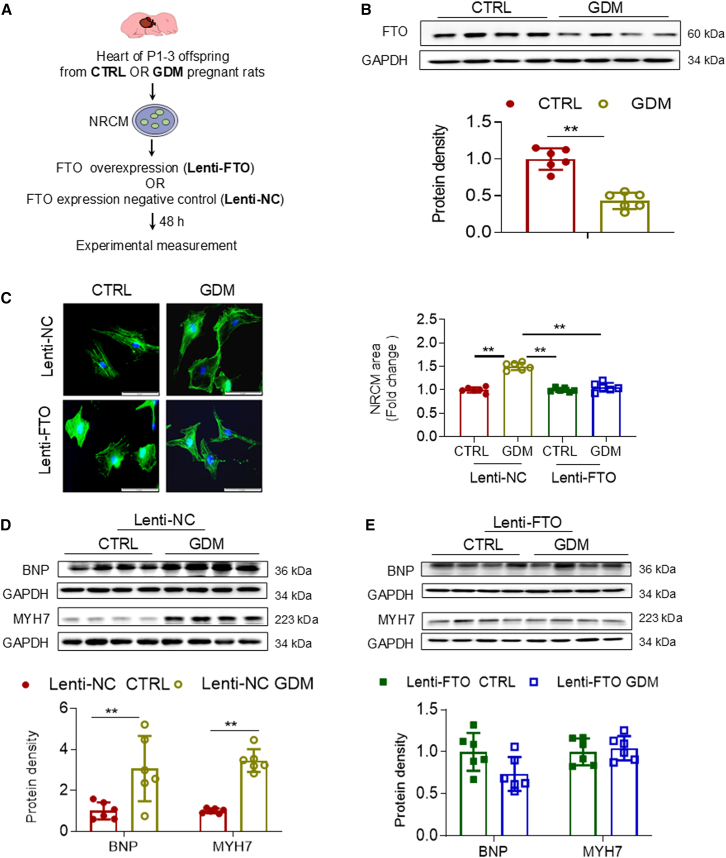
Gene therapeutic targeting of FTO via Lenti-FTO rescues GDM-induced pathological hypertrophy in NRCMs (A) Diagram depicting the isolation of neonatal rat cardiomyocytes (NRCMs) from the hearts of P1–3 offspring from either CTRL or GDM-exposed offspring, followed by Lenti-FTO transfection or negative control treatment (Lenti-NC) for 48 h before experimental measurement. (B) Protein expression of FTO gene was examined using western blotting in NRCMs (CTRL,●; GDM,○). Western blot images are representative samples. Bar graphs reflect the mean ± SD of all six biological samples (*n* = 6 per group). (C) Cardiomyocyte size was measured using immunofluorescence staining of α-actinin in both Lenti-NC- (CTRL,●; GDM,○) and Lenti-FTO (CTRL,▪; GDM,□)-treated NRCMs. DAPI was used to counter stain nuclei. Data are expressed as the averaged surface area of α-actinin-positive NRCMs. The scale bars represent 5 μm. *n* = 6/group. (D and E) Additionally, the protein expression levels of the pathological hypertrophy-related cardiac fetal genes were examined using western blotting analysis in both Lenti-NC- (CTRL,●; GDM,○) (D) and Lenti-FTO-treated (CTRL,▪; GDM,□) (E) NRCMs. Data are expressed as means ± SD, *n* = 6/group. *∗∗p* < 0.01 and *∗p* < 0.05 by Student’s two-tailed unpaired *t* test (B, D, and E) or two-way ANOVA followed by post hoc test (C).

To establish a proof of concept and confirm if the FTO repression is response for GDM-mediated offspring cardiac hypertrophy, neonatal offspring aged 1–3 days from CTRL and GDM-exposed groups were used to isolate neonatal primary cardiomyocytes (NRCMs). Subsequently, the isolated NRCMs were transfected with either FTO lentivirus (Lenti-FTO) or scrambled lentivirus as negative controls (Lenti-NC) (Figure 4A). As depicted in Figure S3, 48 h of treatment with Lenti-FTO significantly enhanced FTO protein expression compared to the CTRL and negative control (Lenti-NC). Consistent with the observations in our *in vivo* GDM-exposed offspring hearts, the expression levels of FTO were markedly decreased in NRCMs isolated from GDM-exposed pups compared to those from CTRL pups (Figure 4B). Immunofluorescent staining for α-actinin revealed an increase in cardiomyocyte size in the Lenti-NC groups following GDM exposure, which was mitigated by Lenti-FTO treatment (Figure 4C). Furthermore, protein levels of BNP and MYH7 in NRCMs isolated from GDM-exposed groups were significantly higher than those from CTRL groups when treated with Lenti-NC (Figure 4D). Treatment with Lenti-FTO abolished these differences in BNP and MYH7 expressions between the GDM and CTRL groups (Figure 4E). These findings suggest that restoration of FTO expression via Lenti-FTO effectively rescues GDM-induced pathological hypertrophy in offspring cardiomyocytes.

### GDM exposure promotes cardiac cellular senescence, inhibits cardiomyocyte proliferation, and induces mitochondrial dysfunction and anti-apoptosis

**Figure 5:**
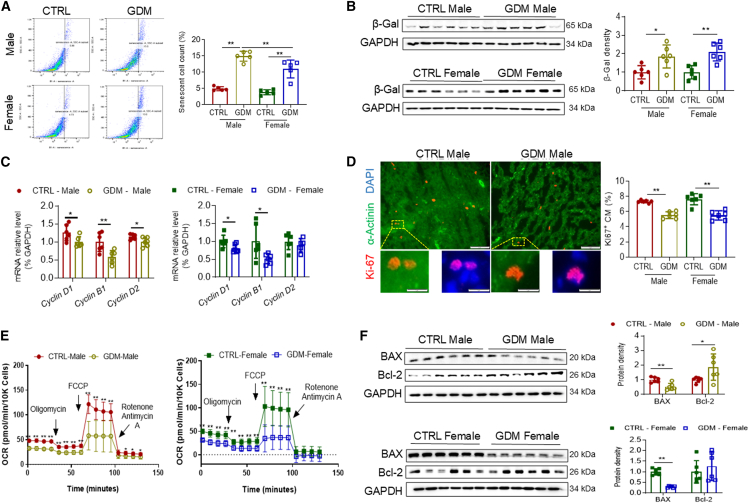
GDM increases cardiac cellular senescence associated with mitochondrial dysfunction and apoptosis resistance in offspring (A) The populations of cardiac senescent cells were measured using flow cytometry in NRCMs isolated from the P1–3 offspring from either CTRL- or GDM-exposed groups. Representative plots show the gating strategy used to identify β-galactosidase (β-gal)-positive NRCMs within the cardiomyocyte population. Quantification of β-gal-positive NRCM population in male offspring (CTRL,●; GDM,○) and female offspring (CTRL,▪; GDM,□). *n* = 5/group. (B) The protein expression levels of β-gal expression in heart tissue at baseline before TAC (P7) were examined using western blotting analysis in male offspring (CTRL,●; GDM,○) and female offspring (CTRL,▪; GDM,□). *n* = 6/group. (C) The relative mRNA levels of the cell cycle regulatory factors, including *Cyclin D1*, *Cyclin B1*, *and Cyclin D2* in myocardium were determined by RT-PCR analysis at baseline before TAC (P7) in male offspring (CTRL,●; GDM,○) and female offspring (CTRL,▪; GDM,□). *n* = 6/group. (D) Ki67 abundance was examined by immunofluorescence staining of cardiac tissues at baseline before TAC (P7). α-Actinin was used to stain myocardium, and DAPI was used to counterstain the nuclei. The scale bars represent 5 μm in the upper and 1 μm in the lower. *n* = 6/group. (E) Mitochondrial oxidative phosphorylation activity was assessed through oxygen consumption rate (OCR) in male offspring (CTRL,●; GDM,○) and female offspring (CTRL,▪; GDM,□). *n* = 7/group. (F) The protein expression levels of the pro-apoptotic marker BAX and anti-apoptotic marker Bcl-2 in the cardiac tissues at baseline before TAC (P7) were determined by western blotting analysis in male offspring (CTRL,●; GDM,○) and female offspring (CTRL,▪; GDM,□). GADPH was used as a loading control. Data are expressed as means ± SD, *n* = 6/group. *∗∗p* < 0.01 and *∗p* < 0.05 by two-way ANOVA followed by post hoc test (A, B, and D) or Student’s two-tailed unpaired *t* test (C, E, and F).

Cellular senescence, characterized by permanent cell-cycle arrest, plays a pivotal role in heart regeneration, cardiac remodeling, atherosclerosis, and heart failure.^22^^,^^23^^,^^24^ We, therefore, examined whether cellular senescence is involved in the GDM exposure-induced cardiac dysfunction in offspring. As illustrated in Figure 5A, flow cytometry analysis revealed that GDM exposure significantly increased the cardiac senescent cell population in both male and female offspring compared to their respective CTRL groups. Notable, the increase in senescent cells was more pronounced in male offspring than in female offspring.

Additionally, protein levels of β-gal, a key marker of cellular senescence, were elevated in GDM-exposed offspring, regardless of sex, relative to their respective CTRL counterparts (Figure 5B). GDM exposure also led to decreased mRNA levels of cell cycle regulatory factors, including *Cyclin D1*, *Cyclin B1*, and *Cyclin D2*, in the neonatal hearts of both male and female offspring compared to CTRL (Figure 5C). Consistent with these findings, Ki67, a widely used proliferation marker, exhibited reduced expression in GDM-exposed hearts of both male and female offspring compared to their respective CTRL (Figure 5D).

Cellular senescence often coincides with mitochondrial dysfunction and altered apoptotic signaling.^25^^,^^26^ To explore this association, we evaluated mitochondrial function and apoptotic profiles in offspring hearts. As shown in Figure 5E, the oxygen consumption rate (OCR) was significantly reduced in the neonatal heart tissues isolated from both male and female offspring of GDM exposed group, compared to their respective CTRL groups. Moreover, GDM exposure decreased the cardiac protein levels of the pro-apoptotic BAX in both male and female offspring and increased the levels of the anti-apoptotic Bcl-2 in male offspring compared to their respective CTRL (Figure 5F). These findings suggest that GDM exposure promotes cardiac cellular senescence, inhibits cardiomyocyte proliferation, and is associated with mitochondrial dysfunction and altered apoptotic signaling pathways.

### Gene therapy targeting FTO rescues GDM-induced cardiac cellular dysfunction

**Figure 6:**
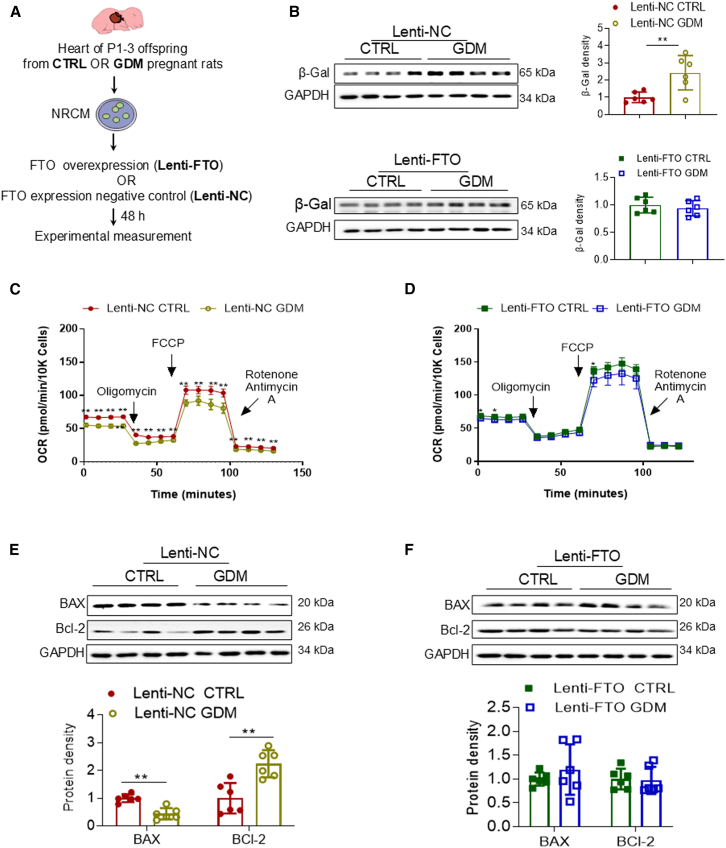
Gene therapeutic targeting of FTO via Lenti-FTO improves GDM-mediated cellular senescence, mitochondrial dysfunction, and apoptosis resistance in NRCMs (A) Diagram depicting the protocol regarding the isolation of NRCMs from P1–3 offspring from either CTRL- or GDM-exposed groups, followed by cardiomyocyte transfection with Lenti-FTO or negative control (Lenti-NC) for 48 h before experimental measurement. (B) The protein expression levels of β-gal were examined using western blotting analysis in both Lenti-NC (CTRL,●; GDM,○) and Lenti-FTO (CTRL,▪; GDM,□) NRCMs. *n* = 6/group. (C and D) Mitochondrial oxidative phosphorylation activity was assessed through oxygen consumption rate (OCR) in Lenti-NC (CTRL,●; GDM,○) (C) and Lenti-FTO-treated (CTRL,▪; GDM,□) (D) NRCMs. *n* = 5/group. (E and F) Additionally, the protein expression levels of the pro-apoptotic marker BAX and anti-apoptotic marker Bcl-2 in NRCMs were determined by western blotting analysis in both Lenti-NC- (CTRL,●; GDM,○) (E) and Lenti-FTO-treated (CTRL,▪; GDM,□) (F) groups. GADPH was used as a loading control. Western blot images are representative samples. Data are expressed as means ± SD, *n* = 6/group. *∗∗p* < 0.01 and *∗p* < 0.05 by Student’s two-tailed unpaired *t* test.

To elucidate whether the FTO repression is response for GDM-induced cellular senescence, mitochondrial deficiency, and altered apoptosis signaling pathways, neonatal primary cardiomyocytes (NRCMs) were freshly isolated from both CTRL and GDM-exposed offspring. Subsequently, the isolated NRCMs were transfected with FTO lentivirus (Lenti-FTO) or scrambled lentivirus as negative controls (Lenti-NC) (Figure 6A). As illustrated in Figure 6B, protein levels of the senescence hallmark, β-gal, in NRCMs isolated from GDM-exposed groups, were significantly higher than those from CTRL groups when treated with Lenti-NC. However, treatment with Lenti-FTO eliminated the differences in β-gal protein expression between the GDM and CTRL groups (Figure 6B, lower). Furthermore, the decrease in mitochondrial OCR induced by GDM exposure (Figure 6C) was partially reversed by Lenti-FTO treatment in isolated NRCMs *in vitro* (Figure 6D). Similarly, the GDM-induced downregulation of BAX and upregulation of Bcl-2 protein levels in NRCMs (Figure 6E) were also rescued by Lenti-FTO treatment in isolated NRCMs *in vitro* (Figure 6F). Taken together, these findings demonstrate that gene therapeutic targeting of FTO can alleviate GDM exposure-induced cardiac cellular senescence, mitochondrial functional deficiency, and anti-apoptosis signaling pathways in offspring.

### Pharmacological inhibition of FTO mimics the detrimental effects of GDM on neonatal cardiomyocytes

**Figure 7:**
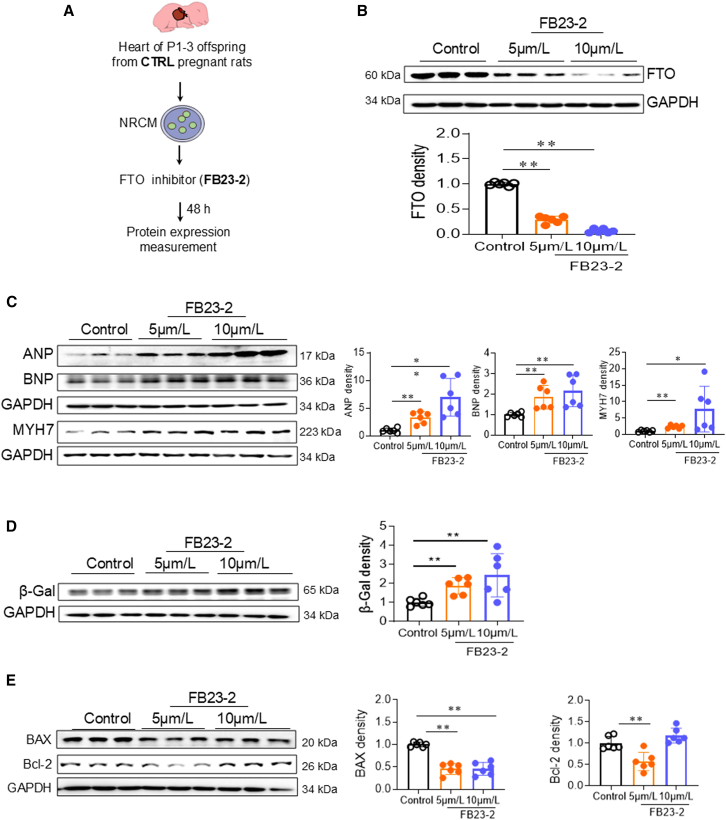
Pharmacological inhibition of FTO promotes cardiomyocyte hypertrophy, cellular senescence, and apoptosis resistance in NRCMs (A) A brief scheme of NRCM isolation from CTRL neonatal (P1–3) offspring, followed by treatment with the FTO inhibitor FB23-2 (5 and 10 μmol/L) for 48 h and then measurement of the cardiac hypertrophy-, cellular senescence-, and apoptosis-associated markers. (B) Treatment with FB23-2 induced a dose-dependent decrease in FTO protein expression in NRCMs. *n* = 6/group. (C) The protein levels of cardiac hypertrophy-associated genes, including ANP, BNP, and MYH7, were examined using western blotting analysis in NRCMs after 48 h of treatment with FB23-2. *n* = 6/group. (D) The protein levels of β-gal were examined using western blotting analysis in NRCMs after 48 h of treatment with FB23-2. *n* = 6/group. (E) The protein levels of apoptosis-associated genes, including BAX and Bcl-2, were examined using western blotting analysis in NRCMs after 48 h of treatment with FB23-2. *n* = 6/group. Western blot images are representative samples. Data are expressed as means ± SD. ∗∗*p* < 0.01 and ∗*p* < 0.05 by one-way ANOVA followed by post hoc test.

To further elucidate whether the detrimental effects of GDM exposure on neonatal offspring heart are associated with FTO repression, NRCMs were freshly isolated from normal pups and treated with a selective FTO inhibitor FB23-2 for 48 h^27^ (Figure 7A). As illustrated in Figure 7B, treatment with FB23-2 resulted in a dose-dependent decrease in FTO protein expression in neonatal cardiomyocytes. This reduced FTO expression was accompanied by an increase in the protein levels of pathological hypertrophy markers, including ANP, BNP, and MYH7, compared to the CTRL group (Figure 7C). Additionally, treatment with FB23-2 induced a dose-dependent increase in the protein expression of β-gal in neonatal cardiomyocytes (Figure 7D). Furthermore, treatment with FB23-2 led to a dose-dependent decrease in the protein expression of BAX (Figure 7E). However, treatment with FB23-2 selectively inhibited Bcl-2 protein expression at the dose of 5 μM but not 10 μM in neonatal cardiomyocytes (Figure 7E). These findings demonstrate that, akin to the detrimental effect of GDM exposure, inhibition of FTO could induce cardiac hypertrophy, cellular senescence, and anti-apoptosis in neonatal cardiomyocytes.

## Discussion

GDM affects approximately 14% of pregnancies worldwide, and emerging evidence indicates its detrimental impact on fetal cardiac development, potentially contributing to later-life heart disease in offspring.^28^^,^^29^ In this study, we investigated the impact of GDM exposure on offspring cardiac function and elucidated the underlying molecular mechanisms. Our results highlight a key role of the m^6^A RNA demethylase FTO in mediating pathological cardiac hypertrophy and dysfunction following prenatal GDM exposure. The major findings of this study are as follows: (1) GDM exposure impaired baseline cardiac function and increased offspring susceptibility to pressure overload-induced heart failure. (2) GDM-exposed offspring exhibited upregulated hypertrophic protein expression and aggravated pathological hypertrophy and fibrosis following TAC. (3) GDM reduced the cardiac expression of the m^6^A demethylase FTO, leading to global and gene-specific RNA hypermethylation. (4) Overexpression of FTO via Lenti-FTO reversed GDM-induced hypertrophy. (5) GDM-exposed offspring exhibited increased cardiac senescence, decreased cardiomyocyte proliferation, reduced mitochondrial oxygen consumption rates, and altered apoptotic signaling, which were alleviated by overexpression of FTO. (6) Pharmacological inhibition of FTO recapitulated the effects of GDM on neonatal cardiomyocytes. Collectively, these findings identify FTO-dependent m^6^A methylation as a critical epigenetic mechanism underlying GDM-induced cardiac dysfunction and suggest that targeting FTO may offer a therapeutic approach to prevent cardiac hypertrophy in offspring.

Epidemiologic and experimental studies have shown that GDM disrupts fetal cardiac development and increases the risk of cardiovascular dysfunction in offspring.^30^ In this study, we used a well-established streptozotocin (STZ)-induced pregnant rat model of GDM in which maternal hyperglycemia was confined to late gestation, closely mimicking human GDM.^31^^,^^32^ Although STZ is cytotoxic to pancreatic β cells, we employed a low dose (50 mg/kg, intraperitoneally) that induces maternal hyperglycemia without elevating offspring glucose levels.^31^^,^^32^ Both our current and prior studies^31^^,^^32^ have demonstrated that this dosage reliably induces maternal hyperglycemia characteristic of GDM without directly elevating blood glucose levels in neonatal offspring. These findings indicate that the observed postnatal cardiac effects are primarily due to maternal GDM exposure rather than direct STZ-induced toxicity. Moreover, previous studies have reported that STZ poorly crosses the placental barrier, thereby minimizing the likelihood of direct fetal exposure or toxicity.^33^ Thus, the observed cardiac phenotypes are attributable to maternal GDM rather than direct STZ toxicity.

We found that GDM exposure reduced fractional shortening (FS%) in P7 neonatal offspring, indicating baseline cardiac dysfunction caused by fetal programming. These results align with clinical observations of impaired cardiac performance in infants born to mothers with GDM.^2^^,^^3^^,^^4^^,^^5^^,^^6^ Moreover, when subjected to TAC, GDM-exposed offspring displayed further reductions in cardiac function, exaggerated hypertrophy, and increased fibrosis, which highlights that GDM predisposes the heart to failure under stress. These findings fill a critical knowledge gap by showing that GDM exposure not only induces basal dysfunction but also impairs the heart’s adaptive response to pathological stress. In line with these functional data, we also observed that GDM exposure increased the protein levels of cardiac hypertrophy markers (ANP, BNP, and MYH7) and exacerbated TAC-induced cardiac hypertrophy, as evidenced by an increased HW/BW ratio, larger cardiomyocyte size, and greater cardiac fibrosis. These results align with recent clinical reports suggesting that GDM exposure is a significant risk factor for the development of myocardial hypertrophy and heart failure in postnatal life.^4^

Epigenetic mechanisms, including DNA methylation, histone modification, and non-coding RNAs, play a pivotal role in the fetal origins of cardiovascular disease.^34^^,^^35^ Previous studies in both humans and animal models have demonstrated that GDM exposure is associated with dysregulation of DNA methylation in offspring.^36^ Our previous study has also shown that GDM exposure is associated with alteration of DNA methylation, leading to the aberrant development of heart ischemia-sensitive phenotype in offspring.^31^ Growing evidence has demonstrated an epitranscriptomic functional role of RNA modifications in fetal development,^17^^,^^34^^,^^37^ which could serve as an additional layer of parental hereditary information. Interestingly, previous studies have demonstrated a key regulatory role of FTO-dependent m6A methylation in the development of heart failure.^38^ Expanding on this, we now found that m6A RNA methylation is also disrupted by GDM. Specifically, GDM exposure increased global m6A levels and altered methylation of the MYH7 gene, with enriched m6A sites at positions 1175 and 3259 in the CDS extending into the 3′ UTR. The potential molecular mechanisms linking increased m6A methylation of MYH7 to impaired cardiac function are not fully understood. We can speculate that the elevated m6A modification on MYH7 mRNA may enhance its stability and translation efficiency, leading to increased MYH7 protein expression and an abnormal shift in the MYH6/MYH7 ratio. This alteration may disrupt sarcomere composition, reduce contractile efficiency, and contribute to cardiac dysfunction. Further studies using site-specific m6A editing or methylation-deficient MYH7 constructs will be required to confirm this causal mechanism.

Importantly, GDM-induced m6A hypermethylation correlated with decreased FTO expression. Emerging evidence indicates that metabolic stressors present in GDM (hyperglycemia, hyperinsulinemia, and oxidative stress) can modulate FTO and other m6A regulators in a context-dependent manner; in some cellular systems, high glucose increases FTO, whereas in others oxidative stress or diabetic conditions are associated with reduced FTO activity, linking metabolic disturbance to m6A dysregulation.^39^^,^^40^ A recent study showed that high glucose levels, which cause oxidative stress, lead to a downregulation of FTO and increased m6A levels.^41^ Consistent with this, our previous study using a similar STZ-induced GDM model demonstrated significantly elevated cardiac oxidative stress in the offspring.^31^ Together, these findings suggest that GDM-related metabolic and oxidative stress repress FTO expression, resulting in m6A-dependent dysregulation of hypertrophic genes.

Reactivation of fetal cardiac genes such as MYH7, ANP, and BNP is a hallmark of pathological hypertrophy. Our current study showed that GDM exposure upregulated these fetal genes in the neonatal heart, implying epigenetic regulatory involvement in cardiac fetal gene programming. The findings that pharmacologic inhibition of FTO with FB23-2 caused significant and dose-dependent increases in the protein levels of these fetal genes, including MYH7, ANP, and BNP, in neonatal cardiomyocytes, suggesting that FTO repression or inhibition may play a role in upregulating cardiac fetal genes and induce cardiac hypertrophy. Furthermore, we demonstrated that GDM exposure-induced increases in cardiomyocyte size and expression of hypertrophic markers (ANP, BNP, and MYH7) were rescued by FTO overexpression via Lenti-FTO. These findings are consistent with previous studies showing that FTO-dependent m6A methylation plays a crucial role in regulating cardiac fetal genes, including MYH7, and in maintaining heart function.^18^^,^^42^ Furthermore, previous studies have suggested that FTO overexpression can inhibit myocyte apoptosis and enhances myocyte contractile function, resulting in the improvement of heart failure.^38^^,^^43^ Taken together, these findings suggest that FTO-dependent m6A methylation is a key epigenetic mechanism in the regulation of cardiac fetal gene expression, and restoring FTO activity may represent a therapeutic strategy to prevent or reverse GDM-induced cardiac dysfunction.

In addition to hypertrophic remodeling, GDM exposure induced cardiomyocyte senescence, proliferation arrest, and fibrosis, which likely contribute to the development of cardiac hypertrophy. Indeed, the present study demonstrated that GDM exposure significantly inhibited the mRNA levels of the cell cycle regulatory factors, including *Cyclin D1*, *Cyclin B1*, and *Cyclin D2*, and reduced the expression levels of the proliferation marker Ki67 in offspring neonatal heart. Furthermore, the present data also showed that GDM exposure enhanced cardiac senescent cell populations associated with an increase in cardiac fibrosis in neonatal offspring heart. Given the fact that cellular senescence is characterized by a cellular proliferative arrest marked by phenotypic alterations, including increased cell size and reduced levels of cyclin-dependent kinase,^13^ our findings strongly suggest that enhanced cardiac cellular senescence could be a key contributor to GDM-induced hypertrophy.

It is well known that cellular senescence plays a significant role in many age-associated diseases.^13^^,^^14^^,^^22^^,^^23^^,^^24^ Our present findings that GDM exposure impairs cardiac development associated with increased cardiac cellular senescence, suggesting that cellular senescence not only plays a key role in age-related diseases but also plays a vital role in development-associated diseases. Currently, the mechanisms underlying GDM exposure-mediated premature cardiac cellular senescence are unclear. A previous study suggests that premature cellular senescence is associated with impaired mitochondrial function.^44^ They reported that umbilical cord mesenchymal stem cells from gestational diabetes have premature senescence phenotypes, characterized by cellular enlargement, senescence-associated β-gal overexpression, and decreased differentiation potential.^44^ This premature senescence phenotype is associated with low mitochondrial activity and reduced expression of the mitochondrial function regulatory genes.^42^ Consistent with this study, our present study demonstrated a significant decrease in the OCR in GDM-exposed neonatal cardiomyocytes. These findings imply that mitochondrial functions in GDM-exposed neonatal cardiomyocytes are impaired, which could be a major cause of decreased cell proliferation and premature cardiac cellular senescence. Given the high energetic demand of the heart, reduced mitochondrial bioenergetics in senescent cardiomyocytes may compromise cardiac performance.^45^

Apoptosis is another key determinant of cardiac cell turnover and homeostasis.^46^ The present findings that GDM exposure attenuated the expression of the pro-apoptotic marker BAX and enhanced the expression of the anti-apoptotic marker Bcl-2 in neonatal offspring hearts suggest the development of an anti-apoptotic phenotype in neonatal hearts. The relationship/interaction between cellular senescence and apoptosis for cell fates has been well documented.^25^ Senescent cells are inherently resistant to apoptosis.^25^^,^^47^ Based on our present findings that GDM exposure-induced cardiac cellular senescence is associated with a decrease in apoptosis, we can speculate that GDM exposure increases myocardial senescence, promotes an anti-apoptotic cellular phenotype that disrupts cellular turnover, impairs mitochondrial function, and results in decreased energy provision, potentially playing a crucial role in cardiac dysfunction in offspring.

Furthermore, we found that the inhibition of FTO with its selective inhibitor FB23-2 induced a dose-dependent regulation of the protein expression of the senescence-associated β-gal and apoptosis-associated markers, including BAX and Bcl-2, in neonatal cardiomyocytes. On the other hand, therapeutic targeting of the FTO with a Lenti-FTO approach reversed the GDM-mediated increase in β-gal expression, decrease in mitochondrial OCR, and apoptosis-related markers (BAX and Bcl-2) in neonatal cardiomyocytes. These findings suggest that FTO-dependent m6A methylation is a common epigenetic mechanism for the regulation of GDM-mediated cardiac hypertrophic fetal genes (MYH7, ANP, and BNP) and the senescence-associated marker β-gal and apoptosis-associated markers. In the heart, m6A demethylase FTO has been shown to be decreased in heart failure patients and animal hearts, and upregulation of FTO could decrease fibrosis and enhance angiogenesis, thereby attenuating cardiac dysfunction after ischemia injury.^18^ Moreover, FTO knockout mice exhibited a worsened cardiac phenotype after TAC surgery, characterized by reduced EF and FS.^38^ These findings suggest that FTO-mediated cellular senescence, accompanied by mitochondrial dysfunction and apoptosis resistance, are critical molecular mechanisms underlying GDM-induced pathological hypertrophy, and therapeutic targeting of FTO could reverse this cardiac hypertrophic phenotype.

Interestingly, male offspring exhibited greater susceptibility to GDM-induced cardiac injury, characterized by higher global m6A methylation and increased cellular senescence compared to females. Sex hormones may influence these effects. Sex hormones can remodel the epigenome and thereby influence gene-regulatory programs, including RNA- and DNA-based epigenetic mechanisms that may affect m^6^A regulators.^48^ Estrogen exerts cardioprotective effects through enhanced antioxidant capacity and anti-senescent signaling via ERα pathways, which could partially protect female offspring from GDM-induced cardiac injury.^49^ However, the offspring in the present study were at pre-pubertal age, suggesting that these sex-dependent differences may also arise from intrinsic chromosomal (XX vs. XY) or early developmental epigenetic programming rather than circulating sex hormones alone. Future studies will explore sex-specific regulatory pathways of m6A enzymes in GDM-induced cardiac programming.

In summary, this study identifies a novel epigenetic mechanism underlying GDM-induced neonatal cardiac hypertrophy and dysfunction. GDM exposure reprograms fetal cardiac gene expression, promotes cellular senescence and anti-apoptotic signaling, and impairs mitochondrial bioenergetics through FTO-dependent m6A methylation. Restoring FTO function mitigates these effects, highlighting FTO as a promising therapeutic target for preventing adverse cardiac outcomes in offspring of diabetic pregnancies.

### Limitations of the study

While this study provides novel mechanistic insights, several limitations should be noted: (1) although the STZ-induced GDM model mimics late-gestational hyperglycemia, it does not fully recapitulate the complex hormonal, inflammatory, and lipid alterations observed in human GDM. Complementary high-fat diet or genetic models may help validate FTO-mediated mechanisms across GDM subtypes. (2) Our analyses focused on early postnatal stages; future longitudinal studies are needed to assess whether FTO-dependent m^6^A dysregulation persists into adulthood and contributes to long-term cardiac pathology. (3) Although we examined molecular and mitochondrial endpoints, future work should include comprehensive cardiomyocyte functional assays (e.g., calcium handling, contractility, and electrophysiology) to strengthen translational relevance. (4) Some experiments had modest sample sizes due to resource constraints; however, results were consistent and statistically robust across replicates. Larger cohorts will improve reproducibility. (5) Finally, while Lenti-FTO overexpression *in vitro* demonstrated mechanistic causality, future studies employing *in vivo* cardiac-specific FTO modulation or small-molecule FTO activators are warranted to confirm therapeutic applicability. Moreover, the precise molecular mechanisms by which FTO-dependent m^6^A methylation mediates cardiac dysfunction in response to GDM exposure require further and more in-depth investigation.

## Resource availability

### Lead contact

Further information and requests for resources and reagents should be directed to and will be fulfilled by the lead contact, DaLiao Xiao (dxiao@llu.edu).

### Materials availability

This study did not generate new unique reagents and animal strain.

### Data and code availability

• All data supporting the findings of this study are available within this paper.
• This study did not generate new datasets and any new code.
• Any additional information reported in this paper is available from the lead contact upon reasonable request.

## Acknowledgments

This work was supported by the 10.13039/100005595Regents of the University of California, Research Grants Program Office, 10.13039/100005188Tobacco-Related Disease Research Program (TRDRP) (grant nos. T29IR0437 to D.X., T34IR8076 to D.X., and T32FT4859 to Y.L.), United States.

## Author contributions

W.Y.: methodology, validation, formal analysis, investigation, and writing – original draft. Y.L.: methodology, validation, formal data analysis, and writing – review and editing. S.J.: methodology, validation, formal analysis, and writing – review and editing. J.T.: methodology and writing – review and editing. S.-W.S.: methodology and writing – review and editing. L.Z.: resources, supervision, and writing – review and editing. D.X.: supervision, resources, conceptualization, project administration, funding acquisition, and writing – review, editing, and finalizing the manuscript. All authors reviewed and approved the final manuscript.

## Declaration of interests

The authors declare no competing interests.

## STAR★Methods

### Key resources table

**Table:** 

REAGENT or RESOURCE	SOURCE	IDENTIFIER
**Biological Samples**		

Pregnant Sprague-Dawley rats	Charles River Laboratory (Portage, MI)	RRID: RGD_10395233

**Oligonucleotides**		

Cyclin B1 Forward: GGTGGAACGACTGTTGGTCT Reverse: TTTCGTGTTCCTGGTGACCC	Integrated DNA Technologies, Inc. (Coralville, Iowa)	N/A
Cyclin D1 Forward: ATTTCCAACCCGCCTTCCAT Reverse: GACAGTCCGCGTCACACTTG	Integrated DNA Technologies, Inc. (Coralville, Iowa)	N/A
Cyclin D2 Forward: GCTCTGTGTGCTACCGACTT Reverse: CACATCGGTGTGGGTGATCT	Integrated DNA Technologies, Inc. (Coralville, Iowa)	N/A
MYH7 1175 Forward: CCATCTCTGACAACGCCTATC Reverse: CCTGGGAGAAGAACGCATAAT	Integrated DNA Technologies, Inc. (Coralville, Iowa)	N/A
MYH7 2257 Forward: GGAGAAGATGGTGTCCCTGC Reverse: AGCCTCTCGGTCATCTCCTT	Integrated DNA Technologies, Inc. (Coralville, Iowa)	N/A
MYH7 3259 Forward: GAACCAGTCCATCCTCATCAC Reverse: CTGCCCTTTGGTGACATACT	Integrated DNA Technologies, Inc. (Coralville, Iowa)	N/A
GAPDH Forward: TGACTCTACCCACGGCAAGTTCAA Reverse: ACGACATACTCAGCACCAGCATCA	Integrated DNA Technologies, Inc. (Coralville, Iowa)	N/A

**Antibodies**		

Rabbit FTO	Cell Signaling Technology	Cat# 45980; RRID: AB_2799294
Mouse GAPDH	Millipore	Cat# MAB374; RRID: AB_2107445
Rabbit WTAP	Cell Signaling Technology	Cat# 56501: RRID: AB_2799512
Rabbit METTL3	Cell Signaling Technology	Cat# 96391; RRID: AB_2800261
Mouse ANP	Santa Cruz Biotechnology	Cat# sc-515701; RRID: AB_3076640
Mouse BNP	Santa Cruz Biotechnology	Cat# sc-271185; RRID: AB_10609757
Mouse MYH7	Santa Cruz Biotechnology	Cat# sc-71575; RRID: AB_2147277
Mouse α-Actinin	Cell Signaling Technology	Cat#69758; RRID: AB_2799767
Rabbit Bcl2	Cell Signaling Technology	Cat#3498S; RRID: AB_1903907
Rabbit BAX	Cell Signaling Technology	Cat# 14796; RRID: AB_271625
Rabbit β-Galactosidase	Cell Signaling Technology	Cat# 27198; RRID: AB_2798940
Rabbit Ki-67	Cell Signaling Technology	Cat# 9129; RRID: AB_2687446
Donkey Anti-Rabbit IgG Alexa Fluor^TM^ 594	Invitrogen	Cat# A21207; RRID: AB_141637
Donkey Anti-Mouse IgG Alexa Fluor^TM^ 488	Invitrogen	Cat# A21202; RRID: AB_141607

**Software and algorithms**		

Images J Version 1.53m	National Institutes of Health	https://www.nih.gov/
GraphPad Prism Version	GraphPad Software	http://www.graphpad.com/

### Experimental model and study participant details

All procedures and protocols in the proposed animal studies were approved by the Institutional Animal Care and Use Committee (IACUC#23-010) of Loma Linda University and followed the guidelines outlined in the National Institutes of Health (US) Guide for the Care and Use of Laboratory Animals. All efforts were made to minimize animal suffering and the number of animals used. Time-dated pregnant Sprague-Dawley rats were purchased from Charles River Laboratories (Portage, MI). Rats were maintained on a 12/12-h light/dark cycle and had *ad libitum* access to water and chow diet.

### Method details

#### Experimental animal model of gestational diabetes mellitus (GDM)

A pregnant rat model of gestational diabetes mellitus (GDM) exposure was established in our laboratory as previously described.^31^^,^^32^ Briefly, pregnant Sprague-Dawley rats (∼3-month-old) were randomly assigned to two groups: saline control (CTRL) and GDM groups. In the GDM group, pregnant rats received an intraperitoneal injection of streptozotocin (STZ) (50 mg/kg, i.p.) (Sigma, USA) on gestational day 12 (E12).^31^^,^^32^ In the CTRL group, pregnant rats received an intraperitoneal injection of the same volume of saline solution on gestational day 12. STZ is commonly used to induce experimental gestational diabetes due to its relatively specific cytotoxic effect on pancreatic β cells to induce maternal hyperglycemia.^50^^,^^51^ Our previous studies using this GDM animal model demonstrated that following STZ injection, the daily blood glucose values of pregnant rats continued to rise and eventually stabilized at 300–400 mg/dL without significantly impacting fetal blood glucose levels.^31^^,^^32^ These blood glucose levels in pregnant rats are comparable to those observed in human cases of uncontrolled severe diabetes during pregnancy.^31^

In the present study, 11 control pregnant rats, and 11 STZ-treated pregnant rats were utilized. A total of 125 offspring from the control group and 118 offspring from the STZ-treated group were delivered. The average litter sizes (mean ± SD) were 11.36 ± 1.6 for the control group and 10.73 ± 1.6 for the STZ treated group. Detailed information regarding litter size per dam, the number of male and female offspring, and their allocation to specific experiments, including TAC studies, were provided in Table S2.

#### Major experimental design of this study

The major experimental designs and procedures are illustrated in Figure 1A and outlined as follows: 1) Pregnant rats received injections of streptozotocin (STZ) or saline (CTRL) on gestational day 12 (E12). The resulting offspring were categorized into either the GDM (STZ) or CTRL groups. 2) Neonatal rat cardiomyocytes (NRCMs) were isolated from pups aged 1–3 days (P1-3) and cultured. These cells were subsequently subjected to various treatments, including transfection with the FTO lentiviral vector, FTO inhibitor (FB23-2), flow cytometry analysis, Seahorse mitochondrial stress tests, and immunofluorescence analysis. 3) Offspring from each experimental group were subjected to transverse aortic constriction (TAC) surgery at postnatal day 7 (P7). Prior to TAC surgery, baseline heart functions were measured, and heart tissues were then collected for Western blot analysis, immunofluorescence staining, RNA extraction for measuring m6A levels, MeRIP-qPCR and qRT-PCR analysis. 4) Fourteen days post-TAC surgery (P21), the offspring’s heart function was reassessed. Cardiac hypertrophy indices, including Masson staining and heart weight/body weight ratio, were also measured.

#### Pressure overload-induced cardiac hypertrophic rat model via transverse aortic constriction (TAC) procedure

Pressure overload is a common adverse stressor to induce a pathological hypertrophic phenotype. The transverse aortic constriction (TAC) procedure is among the most common approaches to evaluate pressure overload-induced pathological hypertrophic cardiomyopathy in rodent animals. In this study, rat offspring were subjected to cardiac pressure overload induced by the TAC procedure, as described previously.^52^^,^^53^ Briefly, the offspring at postnatal day 7 (P7) were anesthetized with inhalation of isoflurane (3% for induction, 1.5% for maintenance) (Vet ONE, USA) mixed with oxygen (1L/min for induction, 0.5L/min for maintenance), followed by ventilation on a rodent ventilator (Kent Scientific, CT, US). Midline sternotomy was performed, and the chest cavity was exposed by sternal interruption at the second intercostal space through blunt dissection. A 5-0 silk suture was placed around the transverse aorta between right innominate artery and left carotid artery. The suture was tied tightly around a 27-gauge blunted needle (0.4 mm, OD), which was placed parallel to the transverse aorta and served as a spacer to regulate diameter of the constricted aorta. The needle was removed quickly after the placement of ligation. The chest cavity was closed layer by layer with 5–0 suture. The sham rats underwent the same surgery but without suture ties around the transverse aorta. After surgery, the offspring were allowed to recover in a single cage for 20 min before returning to their home cages. The TAC procedure was performed on P7, and two weeks later, the offspring underwent pathological cardiac hypertrophic analysis and heart functional measurements.

#### Heart functional measurement by echocardiography

Cardiac function was measured by echocardiography in the offspring rats at postnatal days 7 (P7, baseline before TAC surgery), and 21 (P21, 14 days after TAC surgery) using a Visual Sonics Vevo-3100 imaging system (FUJIFILM VisualSonics Corporation, WA, USA), following previously described.^31^^,^^54^ Briefly, rats were anesthetized with inhalation of 2% isoflurane (Vet ONE, USA), their chests were shaved, and a layer of acoustic-coupling gel was applied onto the thorax. Subsequently, rats were positioned in the left lateral decubitus stance. M-mode recording of the left ventricle (LV) was obtained at the level of the mitral valve in the parasternal view using two-dimensional (2D) echocardiographic guidance, encompassing both short and long axis views. Cardiac function and heart dimensions were assessed via 2D echocardiography. M-mode tracing was used to measure functional parameters including LV end-diastolic dimension (LVEDD), LV end-systolic dimension (LVESD), LV end-diastolic volume (LVEDV) and LV end-systolic volume (LVESV), quantified using the aforementioned primary measurements and accompanying software. The percentage of LV ejection fraction (EF) was calculated as (LVEDV-LVESV)/LVEDV x 100% and the percentage of LV fractional shortening (FS) was calculated as (LVEDD-LVESD)/LVEDD x 100%. Analysis of echocardiographic data was done by Vevo 3100 analysis software. All echocardiographic data were recorded and analyzed blindly with respect to the treatment groups.

All rats were euthanized with an inhalation overdose (5%) of isoflurane, followed by cervical dislocation. Hearts were then removed, flushed with cold PBS, carefully dissected free of large vessels at the heart base and the pericardial tissue under a microscope, snap-frozen in liquid nitrogen and stored at −80°C for subsequent experimental analysis.

#### Masson staining

Fibrosis of the heart was assessed in P21 neonatal pups using the Masson Trichrome Stain kit (StatLab, Catalog No. NC9752152). The Hearts of the pups were fixed overnight at 4°C in 4% paraformaldehyde (PFA), then dehydrated, embedded in an OCT freezing medium, and snap-frozen in cold isopentane. Subsequently, 4 μm cryosections were obtained using a Leica cryostat (Leica, IL, USA), following our established protocols.^27^ Following the manufacturer’s instructions, collagen fibers were stained blue, while viable myocardium was stained red. All slices were scanned using a Keyence BZ-X710 microscope (Keyence, Osaka, Japan). The area of fibrosis and scar size were quantified using ImageJ software.

#### RNA m6A quantification

Global m6A RNA methylation levels in ventricular tissue samples from P7 neonatal pups were measured using the m6A RNA methylation Quantification Kit (EpiQuik, Catalog No. P-9005).^27^ Briefly, total RNA was isolated from ventricular tissue with a purity A260/280 > 1.8. Subsequently, 200 ng of each RNA sample was coated on strip wells, followed by incubation with a capture antibody. After washing with washing buffer, the detection antibody and enhanced solution were sequentially introduced. The signals were then developed by the detection solution. The absorbance of the signals was measured at 450 nm using a microplate reader (Bio-Tek, VT, USA), and the optical density (OD) value was determined by colorimetry, followed by calculation of the total m6A content.

#### Primary neonatal rat cardiomyocytes (NRCMs) isolation

NRCMs were isolated from the hearts of offspring rats aged P1-3 from both the GDM and CTRL groups using the Primary Cardiomyocyte Isolation Kit (Thermo, Catalog No. 88281).^55^ Briefly, after anesthetized with inhalation of 2% isoflurane and decapitated, ventricular tissues were collected, minced, and subjected to digestion with 200 μL of enzyme solution 1 (containing papain) and 10 μL of digestive solution 2 (containing thermolysin) at 37°C for 35 min. The tissue was then gently agitated to primarily achieve a single-cell suspension. The liberated cells were placed in a pre-plated cell culture plate for 1 h to remove non-cardiomyocytes, primarily fibroblasts. Following pre-plating, non-adherent cardiomyocytes were re-suspended and then plated on culture plates, or directly for flow cytometry and Seahorse analysis.

#### Flow cytometry analysis of senescent cell populations

Cellular senescence in isolated cardiomyocytes was evaluated via flow cytometry using the CellEvent Senescence Green Detection kit (Invitrogen, Catalog No. C10840). The procedure involved suspending the cardiomyocytes in PBS, followed by fixation with fixation solution for 10 min and subsequent washing with 1% bovine serum albumin in PBS. Next, each sample received 100 μL of a pre-warmed working solution containing the fluorogenic β-gal substrate, followed by a 2-h incubation at 37°C. The fluorescence signal indicative of senescent cells was analyzed using a MACSQuant Analyzer 10 flow cytometer (Miltenyi Biotec, Bergisch Gladbach, Germany). Flow cytometric analysis was performed using Flowmagic and FlowJo software.

#### Lentiviral FTO gene (Lenti-FTO) transfection in cardiomyocytes

NRCMs from each group of pups were suspended in DMEM with 10% FBS (Gibco, Catalog No. A5256801) and 0.01% Cardiomyocyte Growth Supplement (Thermo, Catalog No. 88281) and plated in 6-well plates (5 × 10^5^ cells per well) or on coverslips in 24-well plates (1 × 10^5^ cells per well). The cardiomyocytes were allowed 24 h of recovery time in culture plates. For over expression of FTO, lentivirus plasmids carrying full-length FTO coding sequence and lentivirus null control plasmids were acquired from GeneCopoeia Inc (Cat# LPPRn18290-Lv105-A00-GS). NRCMs were transfected with the lentiviral vector at 10 or 25 multiplicities of infection (MOI), along with 8 μg/mL polybrene transfection reagent (Millipore Sigma, Catalog No. TR1003G). After 12 h of transfection, the viral transfection solution was replaced with a fresh growth medium.^56^ Subsequent experiments, including fetal gene expression and protein levels, cellular senescence, and mitochondrial functional studies, were conducted after another 48 h of culture.

#### Mitochondrial functional measurement by seahorse analysis

Mitochondrial functional tests were conducted on NRCMs from each experimental group, assessing mitochondrial oxygen consumption rate (OCR) using a Seahorse XF24 flux analyzer (Seahorse Bioscience, MA, USA). Seahorse XF Mitochondrial Stress Test Kits (Seahorse Bioscience, Catalog No. 103015-100) were used. Experiments adhered to the manufacturer’s protocols. Briefly, 5 × 10^4^ cells per well were seeded into a Seahorse XFe24 cell culture microplate with assay medium. For Lenti-FTO transfection experiments, cells were plated with a fresh growth medium and subsequently changed to an assay medium. Following a 1-h incubation in a CO_2_-free 37°C incubator, the basal respiration of NRCMs was measured. To assess ATP-linked OCR, oligomycin (1.5 μM), an ATP synthase inhibitor, was injected. Maximal respiration was determined by adding the uncoupler carbonyl cyanide 4-(trifluoromethoxy) phenylhydrazone (FCCP) (2.5 μM). Finally, to ascertain non-mitochondrial respiration via inhibition of complexes I and III, rotenone (0.5 μM) and antimycin A (AR) (0.5 μM) were injected, respectively.^57^ Each experimental treatment was replicated across 3 wells of each plate as technical replicates, and each experiment included at least 2–3 biological replicates. OCR values were normalized by cell number in each well and expressed as pmol/min/10K cells.

#### Immunofluorescence staining

Immunofluorescence staining was conducted on either 4% PFA-fixed cells or frozen tissue sections. Briefly, following fixation, samples were incubated overnight at 4°C with primary antibodies diluted at 1:100 against FTO, α-Actinin, and Ki-67. Subsequently, Cy3-conjugated secondary antibodies were applied to visualize the staining. Nuclei were counterstained with DAPI (Vectorlabs, Catalog No. H-1200-10). For Wheat Germ Agglutinin (WGA) staining, cardiac tissue sections were stained with FITC-labeled wheat germ agglutinin (WGA, 10 μg/mL) for 30 min at room temperature, followed by PBS washes. Stained samples were observed using a Keyence BZ-X710 microscope (Keyence, Osaka, Japan), and quantification was performed using ImageJ software.

#### Quantitative real-time PCR (qRT-PCR) analysis

Quantitative real-time PCR (qRT-PCR) analysis was performed following previously established protocols.^27^ Total RNA was extracted from freshly isolated left ventricle (LV) tissues obtained from postnatal day 7 (P7) pups using TRIzol Reagent (Sigma-Aldrich, Catalog No. T9424) according to the manufacturer’s instructions. Reverse transcription was carried out using the Maxima First-Strand cDNA Synthesis Kit (Thermo Fisher Scientific, Catalog No. K1622). Following cDNA synthesis, the expression levels of target genes were determined by real-time PCR using SYBR Green Supermix solution (Qiagen, Catalog No. 204143) on a CFX96 Touch Real-Time PCR System (Bio-Rad, USA). The housekeeping gene GAPDH was utilized as an internal control for normalization. Data were analyzed using the 2ˆ^(−ΔΔCT)^ method. Primer sequences used for qRT-PCR are provided in key resources table.

#### Western blotting analysis

Total protein samples were extracted from the LV tissues of P7 pups or NRCMs. Equal amounts of total protein (20 μg) were loaded for each sample. Western blotting analysis was conducted as we previously described.^27^ Briefly, proteins were separated by SDS-PAGE and transferred onto a nitrocellulose membrane. The membrane was then probed with specific primary antibodies according to standard procedures. Band intensities were normalized to the loading control, GAPDH. Data were expressed as relative levels compared to the controls. Detailed information regarding the primary antibodies used in this study is provided in key resources table.

#### Methylated RNA immunoprecipitation and quantitative real-time PCR (MeRIP-qPCR)

The enrichment of m6A-modified RNA fragments was performed using the MeRIP Kit (Epigentek, Catalog No. P-9018) following the manufacturer’s instructions. Total RNA (200 ng) extracted from LV tissues obtained from postnatal day 7 (P7) pups was incubated with Anti-m6A antibody or IgG for 2 h and then washed with a wash buffer. A protein digestion buffer containing proteinase K was added and incubated with the antibody–RNA complex for 15 min to release RNA from the complexes. The RNA binding beads were then resuspended and added to the complex, and the RNA was eluted using an elution buffer. The purified RNA was subjected to qPCR as described above, using MeRIP-qPCR primers listed in key resources table to analyze MYH7 mRNA. The m6A levels were quantified as the percentage of the target RNA in the IP fraction relative to the Input fraction: %Input = 2ˆ^Ct(Input) - Ct(IP)^ × Input Dilution Factor.

### Quantification and statistical analysis

Data are presented as mean ± standard deviation (SD) from at least five independent *in vivo* and *in vitro* experiments (biological replicates); specific group numbers (n) are provided in the figure legends. Statistical analyses were performed using GraphPad Prism software (Version 8, GraphPad Software Inc., San Diego, CA, USA). Differences between two groups were assessed using Student’s *t* test. For evaluating differences among multiple groups, one-way or two-way analysis of variance (ANOVA) was utilized, followed by Tukey’s post-hoc test for multiple comparisons. Statistical significance was considered at *p* < 0.05 for all comparisons.
