# Supplementary material for: FTO-dependent m6A methylation mediates gestational diabetes mellitus-induced offspring cardiac senescent hypertrophy and dysfunction
Source: iScience. 2025 Dec 5;29(1):114311. doi: 10.1016/j.isci.2025.114311 (PMC12774703; doi:10.1016/j.isci.2025.114311)
Supplement: Document S1. Figures S1–S3 and Tables S1 and S2 [file mmc1.pdf]

iScience, Volume *29*

Supplemental information

FTO-dependent m6A methylation mediates gestational diabetes mellitus-induced offspring cardiac senescent hypertrophy and dysfunction

Wansu Yu, Yong Li, Siyi Jiang, Jia Tian, Shu-Wei Sun, Lubo Zhang, and DaLiao Xiao

Supplementary Materials

FTO dependent m6A methylation mediates gestational diabetes mellitus-induced offspring cardiac senescent hypertrophy and dysfunction

Wansu Yu[1,2], Yong Li[1], Siyi Jiang [1,3], Jia Tian[1], Shu-Wei Sun[1], Lubo Zhang[1], and DaLiao Xiao[1]

[1] Lawrence D. Longo, MD Center for Perinatal Biology, Department of Basic Sciences, Loma Linda University School of Medicine, Loma Linda, California, USA.
[2] Department of Geriatrics, Guangzhou First People's Hospital, School of Medicine, South China University of Technology, Guangzhou, China.
[3] Department of Hematology, The Third Xiangya Hospital of Central South University, Changsha, China.

Running title: Epigenetic role of FTO in GDM-induced cardiac developmental defect

Corresponding author:

Daliao Xiao, PhD
Lawrence D. Longo, MD Center for Perinatal Biology
Department of Basic Sciences
Loma Linda University School of Medicine
Loma Linda, CA, USA 92350
Tel: 909-558-4325
Fax: 909-558-4029
E-mail: Dxiao@llu.edu

Supplementary Table S1. Effect of TAC procedure on left ventricular parameters in offspring at P21 compared to sham group.

Animal groups	CRTL - Sham (P21) (N＝13)	CRTL - 14 Days after TAC (P21) (N＝13)
EF (%)	71.433±1.503	65.464±2.445 *
FS (%)	41.554±1.051	36.789±1.876 *
IVSd (mm)	0.923±0.040	1.458±0.052 **
IVSs (mm)	1.627±0.025	2.299±0.042 **
LVIDd (mm)	4.951±0.106	5.631±0.309 *
LVIDs (mm)	2.950±0.105	3.601±0.270
LVPWd (mm)	0.958±0.051	1.515±0.076 **
LVPWs (mm)	1.655±0.061	2.304±0.090 **
LV Vol d (μL)	118.131±5.441	170.400±19.529 *
LV Vol s (μL)	34.416±2.773	60.180±10.263 *
SV (μL)	83.715±3.596	110.220±10.423 *

Note: Data are expressed as means ± SD, **P < 0.01 and *P<0.05 by Student's two-tailed unpaired *t*-test.

Supplementary Table S2. Summary of Offspring Distribution and Experimental Allocation

Experiment							
Mother-Group	Total Litter Size	P1-3 Isolated NRCM	P1-3 Isolated NRCM (Male/Female)	P7 Baseline (Male/Female)		P21 Post-TAC (Male/Female)	
				Collected heart tissues	TAC surgery	Collected heart tissues	Death
Control-1	14				8/6	6/5	2/1
Control-2	11			5/6			
Control-3	12			7/5			
Control-4	13			6/7			
Control-5	10	10					
Control-6	9		4/5				
Control-7	13	13					
Control-8	10		5/5				
Control-9	11	11					
Control-10	12	12					
Control-11	10		3/2	2/2	0/1	0/1	0/0
GDM-1	10				6/4	4/3	2/1
GDM-2	9				5/4	4/3	1/1
GDM-3	11			6/5			
GDM-4	13			6/7			
GDM-5	12			6/6			
GDM-6	9	9					
GDM-7	11	11					
GDM-8	8	8					
GDM-9	11		5/6				
GDM-10	13		7/6				
GDM-11	11			2/2	2/5	2/4	0/1

Total							
Mother-Group	Total Litter Size	P1-3 Isolated NRCM	P1-3 Isolated NRCM (Male/Female)	P7 Baseline (Male/Female)		P21 Post-TAC (Male/Female)	
				Collected heart tissues	TAC surgery	Collected heart tissues	Death
Control	125	46	12/12	20/20	8/7	6/6	2/1
GDM	118	28	12/12	20/20	13/13	10/10	3/3

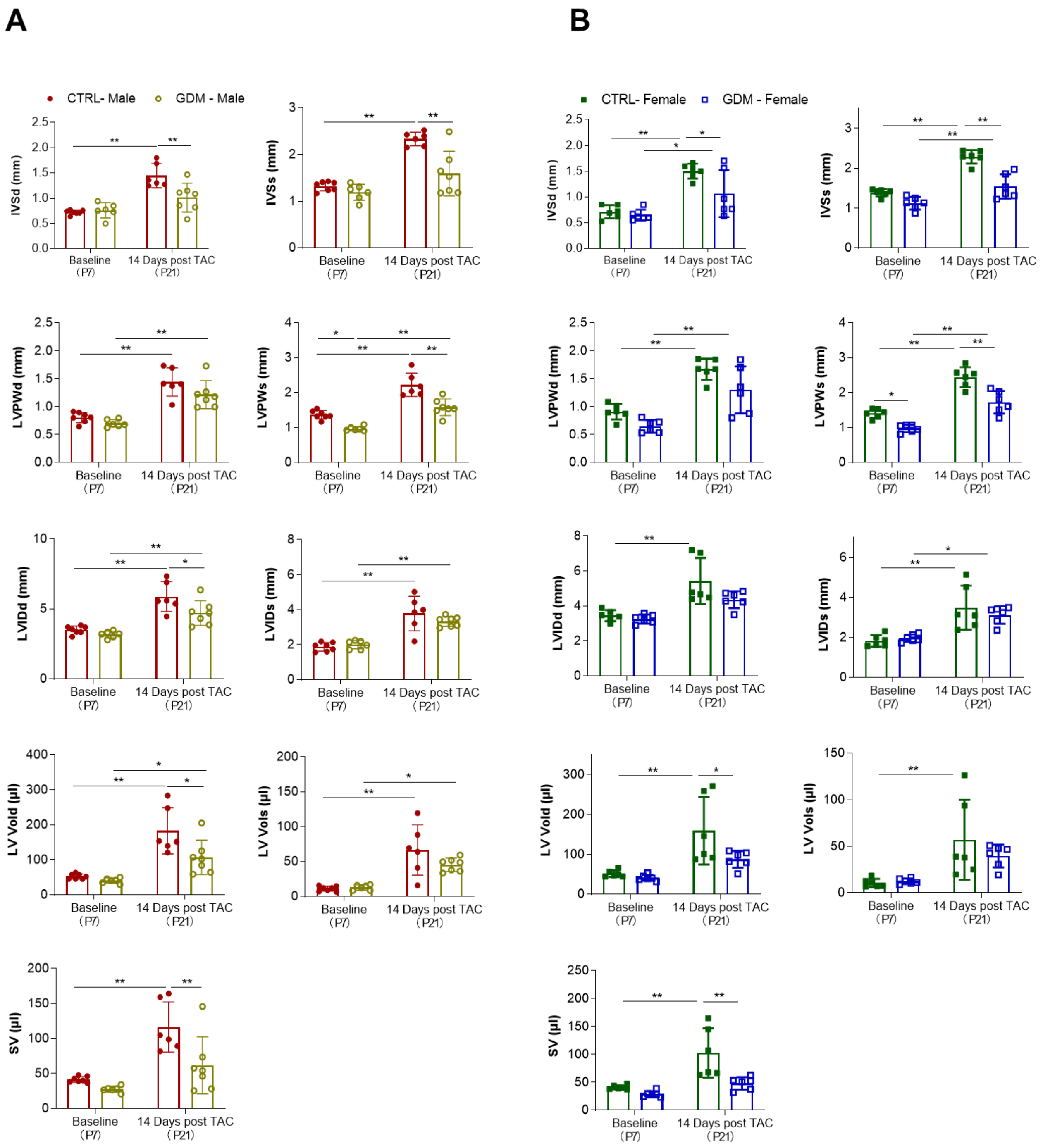

Supplementary Fig. 1

Supplementary figure 1 legend. Effect of GDM on heart function in offspring

Cardiac performance was examined using two-dimensional echocardiography at baseline before TAC (P7) and 14 days post TAC (P21) in male offspring **(A)** (CTRL,●; GDM,○) and female offspring **(B)** (CTRL,■; GDM,□). Data are expressed as means ± SD, ***P < 0.01 and *P<0.05* by two-way ANOVA followed by post hoc test. n = 7/group for Baseline CTRL male group and 14 days post TAC GDM male group, n = 6/group for Baseline GDM male group, 14 days post TAC CRTL male group and female groups.

Abbreviations: IVSd/IVSs, inter ventricular septum thickness at diastolic/systolic phase; LVPWd/LVPWs, left ventricular posterior wall thickness at diastole/systole phase; LVIDd/LVIDs, left ventricular internal diameter at diastolic/ systolic phase; LV Vold/LV Vols, left ventricular volume at diastolic/ systolic phase, SV, stroke volume.

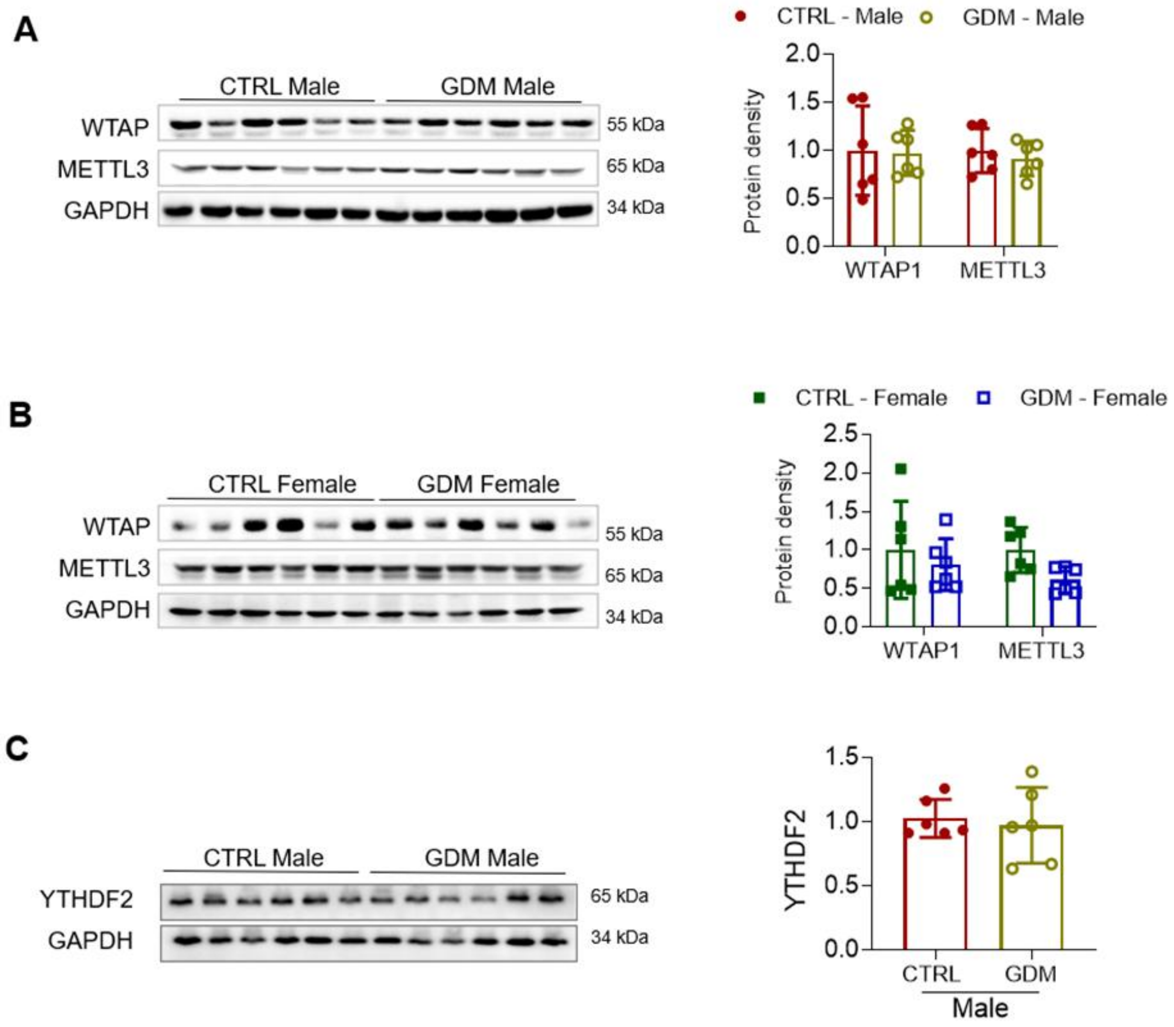

Supplementary Fig. 2

Supplementary figure 2 legend. GDM exposure has not only no effects on the protein levels of WTAP and METTL3 in neonatal offspring heart, but also no effects on the protein levels of YTHDF2 in neonatal male offspring hearts.

The protein expression levels of WTAP and METTL3 in cardiac tissues at baseline before TAC (P7) were examined using western blotting in male offspring (CTRL,●; GDM,○) (**A**) and female offspring (CTRL,■; GDM,□) (**B**). Additionally, the protein expression levels of YTHDF2 in cardiac tissues at baseline before TAC (P7) were also examined using western blotting in male offspring (CTRL,●; GDM,○) (**C**). The blots against GAPDH served as loading controls. Data are expressed as means ± SD, n = 6/group. No significance by Student's two-tailed unpaired *t* test.

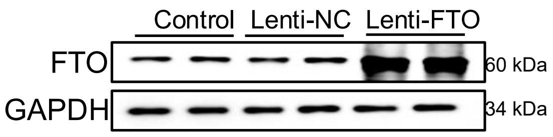

Supplementary Fig. 3

Supplementary figure 3 legend. FTO lentivirus transfection increases the protein expression of FTO in NRCMs

The protein levels of FTO were examined using western blotting analysis in neonatal rat cardiomyocytes (NRCMs). Control group displayed baseline FTO expression. Transfection with negative control lentivirus (Lenti-NC) had no effect on FTO protein expression. However, transfection with lenti-FTO FTO resulted in increased FTO levels compared to the control group after 48 hours of transfection.
